# Prediction of Six-Month Glycemic Status in Type 2 Diabetes: Development and Internal Validation of a Multivariable Model

**DOI:** 10.3390/diagnostics16193201

**Published:** 2026-10-02

**Authors:** Andreea Diana Igna, Bianca-Lăcrimioara Petca, Paula-Alexandra Popovici, Ioana Moga, Titus David Moga, Timea Claudia Ghitea, Corina Cinezan, Mihaela Simona Popoviciu

**Affiliations:** 1 Doctoral School of Biological and Biomedical Sciences, University of Oradea, 1 University Street, 410087 Oradea, Romania; igna.andreeadiana@student.uoradea.ro (A.D.I.); criste.biancalacrimioara@student.uoradea.ro (B.-L.P.); cipleu.paulaalexandra@student.uoradea.ro (P.-A.P.); 2 Department of Morphological Disciplines, Faculty of Medicine and Pharmacy, University of Oradea, 410028 Oradea, Romania; mgaioana@gmail.com (I.M.); titusmoga@gmail.com (T.D.M.); 3 Pharmacy Department, Faculty of Medicine and Pharmacy, University of Oradea, 1 University Street, 410087 Oradea, Romania; 4 Department of Medical Disciplines, Faculty of Medicine and Pharmacy, University of Oradea, 410087 Oradea, Romania; ccinezan@uoradea.ro; 5 Department of Preclinical Disciplines, Faculty of Medicine and Pharmacy, University of Oradea, 410028 Oradea, Romania; mihaela.popoviciu@didactic.uoradea.ro

**Keywords:** type 2 diabetes mellitus, HbA1c, glycemic status, prediction model, internal validation, bootstrap, calibration, decision-curve analysis, TRIPOD

## Abstract

**Background:** Baseline HbA1c is expected to dominate prediction of subsequent HbA1c status; therefore, the clinically relevant question is whether additional routinely available variables provide reproducible incremental value. **Methods:** This retrospective longitudinal study used 809 records from adults with type 2 diabetes receiving routine outpatient care. Six-month HbA1c was available for 808 participants. The primary outcome was six-month glycemic status (HbA1c < 7.0%). An HbA1c-only benchmark and three fixed blockwise logistic models were compared in the same complete-case cohort (*n* = 806). Internal validation used 1000 bootstrap resamples and reported optimism-corrected AUC, Brier score, calibration intercept, and calibration slope. Correlations, variance inflation factors (VIFs), nonlinear effects, decision-curve analysis, and a sensitivity analysis restricted to baseline HbA1c ≥ 7.0% were evaluated. **Results:** At baseline, 215 participants had HbA1c < 7.0%; 179 maintained this status, 206 newly reached <7.0%, 36 moved above the threshold, and 387 remained ≥7.0%. In complete cases, the optimism-corrected AUC was 0.868 (95% CI 0.844–0.892) for HbA1c alone and 0.870 (95% CI 0.848–0.893) for the full model. The corrected AUC difference between the full and HbA1c-only models was 0.001 (95% CI of −0.011 to 0.011). For the full model, the corrected Brier score was 0.146, the calibration intercept was 0.001, and the calibration slope was 0.925. VIFs were low (maximum of 1.33), although the TyG coefficient reversed direction after adjustment and was not interpreted causally. In participants with baseline HbA1c ≥ 7.0% (*n* = 592 complete cases), corrected AUCs were 0.850 for HbA1c alone and 0.849 for the full model. **Conclusions:** Baseline HbA1c accounts for nearly all discriminative performance. Added biomarkers and baseline therapeutic classes provide little internally validated incremental value, and the model should not be used for treatment decisions before external validation and prospective evaluation of clinical impact.

## 1. Introduction

Glycated hemoglobin (HbA1c) reflects average glycemic exposure over approximately two to three months and is central to monitoring of long-term glycemic control because it is less affected by short-term glucose fluctuations than a single fasting glucose measurement. HbA1c is also associated with the risk of microvascular complications and is the principal metric used to assess therapeutic response. A value below 7.0% is a common population-level benchmark for many nonpregnant adults, but guideline-recommended targets must be individualized according to age, comorbidity, hypoglycemia risk, disease duration, and patient preferences [1,2,3,4].

Subsequent HbA1c is necessarily related to baseline HbA1c. Accordingly, a prediction study is clinically informative only if additional predictors improve performance beyond an HbA1c-only benchmark and if the model is adequately validated. Treatment allocation, adherence, lifestyle, socioeconomic circumstances, and treatment changes during follow-up may also influence six-month glycemia and can confound associations observed in routine care.

Routinely available cardiorenal variables—including the triglyceride–glucose (TyG) index, urinary albumin-to-creatinine ratio (UACR), estimated glomerular filtration rate (eGFR), C-reactive protein (CRP), and Fibrosis-4 (FIB-4) index—may describe metabolic complexity, but their incremental predictive value after baseline HbA1c is uncertain. Because TyG is calculated from fasting glucose and triglycerides, coefficient direction and stability require explicit correlation and collinearity assessment.

The present analysis therefore focuses on transparent model development rather than claiming an immediately actionable risk tool. It compares an HbA1c-only model with sequential predictor blocks fixed for the revised analysis, quantifies optimism through bootstrap validation, reports calibration beyond the Hosmer–Lemeshow test, examines clinical utility using decision-curve analysis, and provides the complete prediction equation in accordance with TRIPOD reporting principles [5,6,7,8].

The primary objective was to predict six-month glycemic status (HbA1c < 7.0%) in adults with type 2 diabetes receiving routine outpatient care. Because the cohort included patients who were already below 7.0% at baseline, a sensitivity analysis evaluated participants with baseline HbA1c ≥ 7.0%, for whom the follow-up outcome more closely represents newly attained target status.

## 2. Materials and Methods

### 2.1. Study Design and Population

This retrospective longitudinal observational study was conducted at a tertiary diabetes center during the period January 2016–December 2021. The source population comprised 811potentially eligible adult records. The screening flow was: 811records assessed two records that did not meet the requirements for baseline cohort inclusion excluded, and 809 records included in the baseline cohort. Eligibility required adult age, documented type 2 diabetes mellitus, a baseline clinical and laboratory assessment, and an intended follow-up assessment at approximately six months. Follow-up HbA1c was considered eligible when obtained within 5–7 months after baseline. Individual assessment dates were not available in the analytical dataset; therefore, the median, interquartile range, and observed minimum–maximum follow-up interval could not be calculated. One record lacked six-month HbA1c and was excluded from outcome analyses, leaving 808 participants. Two additional participants lacked baseline UACR, leaving 806 complete cases for comparisons involving all Model 3 predictors. Non-diabetic controls were not included because the model addresses glycemic status conditional on established type 2 diabetes and the modeled outcome is not defined for a non-diabetic comparison population. The participant flow is provided in Supplementary Figure S1; all bracketed fields must be completed from institutional records before submission.

### 2.2. Clinical and Laboratory Assessment

Baseline variables comprised age, sex, diabetes duration, environment, occupational status, body mass index (BMI), HbA1c, fasting plasma glucose, lipid profile, serum creatinine, eGFR, UACR, CRP, FIB-4, TyG, cardiovascular and microvascular comorbidities, and antidiabetic drug classes. Women were coded separately from men in all regression analyses. Family history, self-reported ethnicity, medication adherence, diet, physical activity, and detailed socioeconomic measures were not available. Baseline therapy was coded as use versus non-use of insulin, biguanides, sulfonylureas, DPP-4 inhibitors, GLP-1 receptor agonists, and SGLT2 inhibitors.

### 2.3. Outcome Measures

The primary outcome was six-month glycemic status, defined as HbA1c < 7.0% at follow-up. This threshold was used as a reproducible cohort-level benchmark and not as a universal individualized treatment target. Baseline-by-follow-up status was cross-tabulated to distinguish maintenance of HbA1c < 7.0% from newly attained status. A sensitivity analysis was restricted to participants with baseline HbA1c ≥ 7.0%. Baseline-to-follow-up changes were treated as descriptive because grouping by the final HbA1c value creates mathematical coupling and may amplify regression-to-the-mean effects.

### 2.4. Statistical Analysis

Continuous variables are presented as mean ± standard deviation or median (interquartile range), and categorical variables as number (percentage). Welch’s t test, Mann–Whitney U test, or the chi-square test was used for descriptive between-group comparisons, as appropriate. Univariable logistic regressions were retained only as exploratory descriptions and were not used to screen predictors for multivariable modeling.

Predictor blocks were fixed for the revised analysis based on clinical and conceptual considerations: HbA1c-only; Model 1 (age, sex, diabetes duration, baseline HbA1c, and BMI); Model 2 (Model 1 plus TyG, eGFR, ln[UACR+1], FIB-4, and ln[CRP+1]); and Model 3 (Model 2 plus the six baseline antidiabetic drug classes). All models were compared in the same 806 complete cases. Fasting plasma glucose was not entered together with TyG because glucose is a component of the TyG formula; an alternative model replacing TyG with fasting glucose was examined as a sensitivity analysis. The revised block structure was not prospectively registered and should not be interpreted as having been specified before examination of the outcome data.

Internal validation used 1000 bootstrap resamples. The full modeling procedure was repeated in each resample, and optimism was estimated as the difference between performance in the bootstrap sample and performance when the bootstrap-fitted model was applied to the original cohort. We report apparent and optimism-corrected AUC, Brier score, calibration intercept, and calibration slope with percentile-95% confidence intervals. Paired bootstrap resampling with 500 replicates was used for AUC differences, and 500 resamples were used for the baseline-HbA1c ≥ 7.0% sensitivity cohort. The Youden threshold, sensitivity, and specificity are reported as exploratory because a clinically justified intervention threshold was not prespecified.

Pearson and Spearman correlations were calculated among baseline HbA1c, fasting glucose, triglycerides, and TyG. Multicollinearity was assessed using variance inflation factors. Natural cubic splines with four degrees of freedom were compared with linear terms to assess nonlinearity. Coefficient stability, including the direction of the TyG coefficient, was examined across bootstrap samples.

Clinical utility was explored with decision-curve analysis across threshold probabilities from 0.10 to 0.80. Because the modeled positive outcome was follow-up HbA1c < 7.0%, the clinically relevant action was defined as intensified monitoring and/or treatment review for patients predicted not to achieve this threshold; the plotted risk for this action was therefore the predicted probability of non-achievement (1 − *p*). In this framing, “treat all” denotes intensified monitoring/treatment review for every patient, whereas “treat none” denotes no intensified monitoring/treatment review for any patient. False-positive classifications would lead to unnecessary additional monitoring or treatment review, whereas false-negative classifications could miss patients who might benefit. No intervention threshold was prespecified, so these curves remain exploratory. The analysis was not prospectively registered. With 384 outcome events in the 806-patient complete-case cohort and 16 predictor parameters in Model 3, the observed events-per-parameter ratio was 24.0; this post hoc description does not replace a prospective sample-size calculation [8].

### 2.5. Ethical Considerations

The study was conducted in accordance with the Declaration of Helsinki and was approved by the Ethical Committee of the Faculty of Medicine and Pharmacy, University of Oradea (approval code 43; 31 October 2025). Informed consent was obtained from all participants, as stated in the institutional ethics declaration.

## 3. Results

### 3.1. Cohort Flow and Baseline Characteristics According to Six-Month Glycemic Status

Among 808 participants with an available six-month HbA1c, 385 (47.6%) had HbA1c < 7.0% and 423 (52.4%) had HbA1c ≥ 7.0%. At baseline, 215 participants were already below 7.0%: 179 maintained this status and 36 moved to ≥7.0%. Among 593 participants with baseline HbA1c ≥ 7.0%, 206 attained <7.0% and 387 remained ≥7.0% at follow-up (Table 1).

The outcome cohort included 378 women (46.8%) and 430 men (53.2%). A total of 399 participants were non-obese (BMI < 30 kg/m^2^); 198 (49.6%) had HbA1c < 7.0% at follow-up, compared with 187 of 409 participants with obesity (45.7%; *p* = 0.298). Thus, non-obese adults were well represented, and obesity status was not significantly associated with six-month glycemic status.

Participants with HbA1c < 7.0% at follow-up had lower baseline HbA1c (7.00 ± 0.77% vs. 8.81 ± 1.62%; *p* < 0.001), fasting plasma glucose (164.24 ± 50.19 vs. 187.44 ± 61.38 mg/dL; *p* < 0.001), and TyG (9.32 ± 0.58 vs. 9.45 ± 0.67; *p* = 0.003). Age differed only marginally (64.04 ± 9.83 vs. 62.66 ± 10.20 years; *p* = 0.050) (Table 2).

Baseline insulin use was less frequent among participants with follow-up HbA1c <7.0% (17.7% vs. 33.1%; *p* < 0.001), and SGLT2 inhibitor use showed a small unadjusted difference (31.4% vs. 38.3%; *p* = 0.049). These associations describe baseline treatment patterns and must not be interpreted as comparative treatment effects.

Complete-case modeling used 806 participants because follow-up HbA1c was unavailable for one record and baseline UACR was unavailable for two records. Missingness for all other Model 3 predictors was zero. Participant flow is shown in Supplementary Figure S1. The Supplementary Table S1 show the correlation and collinearity diagnostics, and the Supplementary Table S2 the sensitivity analysis restricted to baseline HbA1c ≥ 7.0% (n = 592 complete cases; 206 events).

### 3.2. Baseline-to-Follow-Up HbA1c Status

The baseline-by-follow-up table demonstrates that the primary endpoint combines two clinically distinct states: maintenance of HbA1c < 7.0% and newly attained HbA1c < 7.0%. Consequently, the endpoint is referred to throughout as six-month glycemic status, while newly attained status is evaluated separately among participants with baseline HbA1c ≥ 7.0%.

Changes in HbA1c, fasting glucose, and TyG stratified by the final HbA1c category were removed as inferential comparisons because the grouping variable is mathematically linked to those changes. Any remaining change summaries should be interpreted descriptively and not as evidence that group membership caused metabolic improvement.

**Figure 1:**
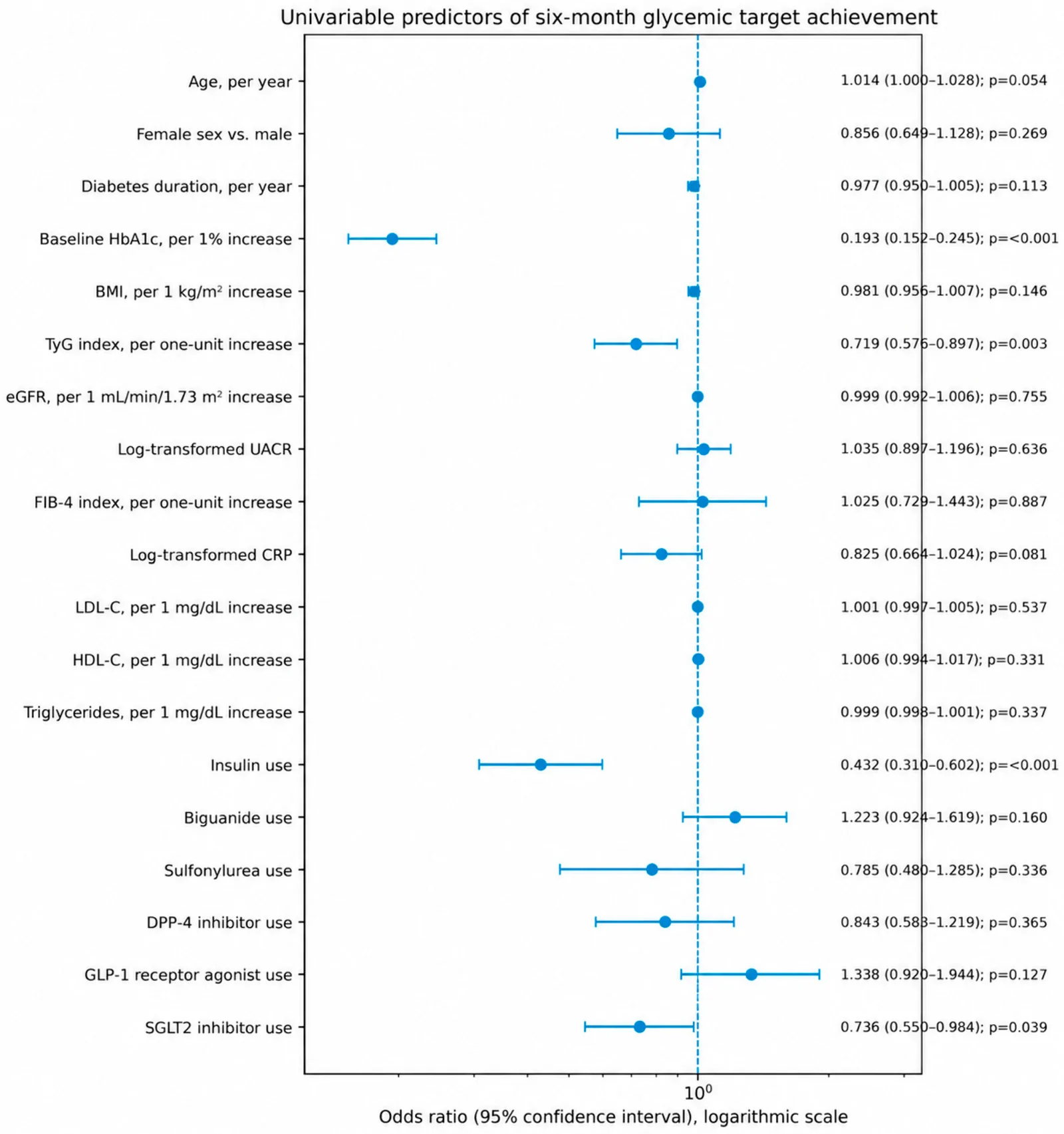
Exploratory univariable associations between baseline variables and six-month HbA1c < 7.0%. These associations were not used to select predictors for multivariable modeling and should not be interpreted causally. Points represent odds ratios, and horizontal bars indicate 95% confidence intervals. The vertical dashed line marks an odds ratio of 1, indicating no association. The horizontal axis uses a logarithmic scale.

The sensitivity cohort contained 592 complete cases with baseline HbA1c ≥ 7.0%, of whom 206 attained HbA1c < 7.0% at follow-up (Figure 1).

### 3.3. Exploratory Univariable Associations

Exploratory univariable logistic regressions described unadjusted associations with six-month HbA1c < 7.0%. All predictors in the fixed blocks were retained in the multivariable models, irrespective of univariable statistical significance.

Baseline HbA1c was the strongest univariable predictor: each 1-percentage-point increase was associated with lower odds of HbA1c < 7.0% at follow-up (OR of 0.189, 95% CI 0.148–0.241; *p* < 0.001).

Higher TyG was inversely associated with the outcome before adjustment (OR 0.717, 95% CI 0.575–0.895; *p* = 0.003), and baseline insulin use was also associated with lower odds (OR 0.434, 95% CI 0.311–0.604; *p* < 0.001).

Baseline SGLT2 inhibitor use had a modest inverse unadjusted association (OR 0.738, 95% CI 0.552–0.988; *p* = 0.041). This likely reflects treatment allocation according to baseline disease and cardiorenal risk and is not an estimate of drug efficacy.

Age (OR 1.014 per year; *p* = 0.051) and ln(CRP+1) (OR 0.825; *p* = 0.081) showed borderline univariable associations (Table 3).

**Figure 2:**
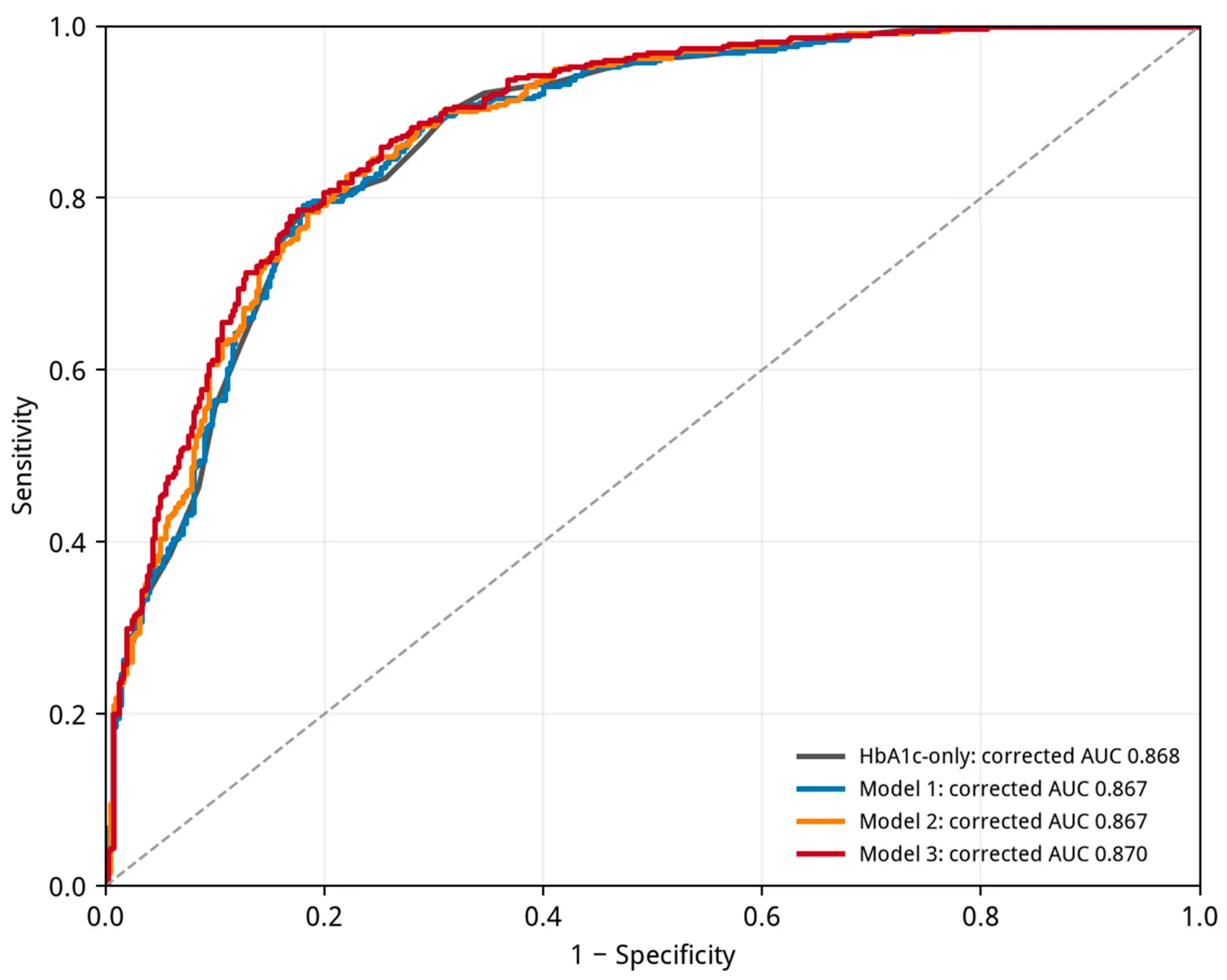
Receiver operating characteristic curves for the HbA1c-only model and Models 1–3 in the common complete-case cohort (*n* = 806). Legend values are optimism-corrected AUCs from 1000 bootstrap resamples. The curves show minimal incremental discrimination beyond baseline HbA1c alone. The diagonal dashed line represents chance-level discrimination (AUC = 0.5).

Sex, diabetes duration, BMI, eGFR, albuminuria, FIB-4, lipid variables, and the remaining therapeutic classes were not significantly associated in univariable analyses (Figure 2).

Because univariable screening may produce unstable and biased prediction models, these *p* values were not used as entry criteria. The predictor blocks and rationale are specified in Section 2.4.

### 3.4. Multivariable Model Development and Internal Validation

Four fixed logistic models were fitted in the same 806 complete cases. Model development used the variables in the fixed blocks rather than univariable *p*-value screening.

The HbA1c-only model had an apparent AUC of 0.869 and an optimism-corrected AUC of 0.868 (95% CI 0.844–0.892). Model 1 had a corrected AUC of 0.867 (95% CI 0.842–0.889), indicating no gain from adding age, sex, diabetes duration, and BMI; its AIC (728.14) was also higher than that of the HbA1c-only model (722.03).

Model 2 had an apparent AUC of 0.875 but a corrected AUC of 0.867 (95% CI 0.847–0.891). The biomarker block did not improve model fit (likelihood-ratio χ^2^ = 7.74, 5 df; *p* = 0.171).

Model 3 had an apparent AUC of 0.881 and an optimism-corrected AUC of 0.870 (95% CI 0.848–0.893). Although the therapeutic block improved in-sample likelihood (likelihood-ratio χ^2^ = 14.84, 6 df; *p* = 0.022), its corrected AUC gain over Model 2 was only 0.002 (95% CI −0.006 to 0.008), and the corrected gain over HbA1c alone was 0.001 (95% CI −0.011 to 0.011). Thus, improved model fit did not translate into a clinically meaningful or statistically precise improvement in discrimination.

In Model 3, baseline HbA1c remained dominant (adjusted OR 0.184 per 1% increase, 95% CI 0.143–0.238; *p* < 0.001). Baseline insulin use was associated with lower odds of six-month HbA1c < 7.0% (adjusted OR 0.563, 95% CI 0.357–0.887; *p* = 0.013), but this association is susceptible to confounding by indication and unmeasured treatment changes.

TyG changed from an inverse univariable association to a positive adjusted association (adjusted OR 1.412, 95% CI 1.042–1.914; *p* = 0.026). Pearson correlations were 0.302 between HbA1c and TyG, 0.548 between fasting glucose and TyG, and 0.795 between triglycerides and TyG. VIFs were 1.18 for HbA1c and 1.12 for TyG (maximum VIF among predictors, 1.33), which does not support severe conventional multicollinearity. The TyG coefficient was positive in 99.0% of bootstrap samples; nevertheless, because its sign reversed after adjustment and TyG added negligible validated performance, it is interpreted as a model-dependent suppression effect rather than a directly interpretable biological association (Table 4).

Natural cubic spline tests provided no evidence of departure from linearity for HbA1c (*p* = 0.901), TyG (*p* = 0.195), age (*p* = 0.678), diabetes duration (*p* = 0.838), or BMI (*p* = 0.897). Replacing TyG with fasting glucose produced a similar apparent AUC (0.880) and did not materially change conclusions.

For Model 3, the optimism-corrected Brier score was 0.146 (95% CI 0.132–0.161), with a calibration intercept of 0.001 (95% CI −0.193 to 0.197) and calibration slope of 0.925 (95% CI 0.789–1.064). The exploratory Youden threshold was 0.551, with apparent sensitivity of 78.6% and specificity of 82.5%; these threshold-dependent estimates were not selected for clinical use.

**Figure 3:**
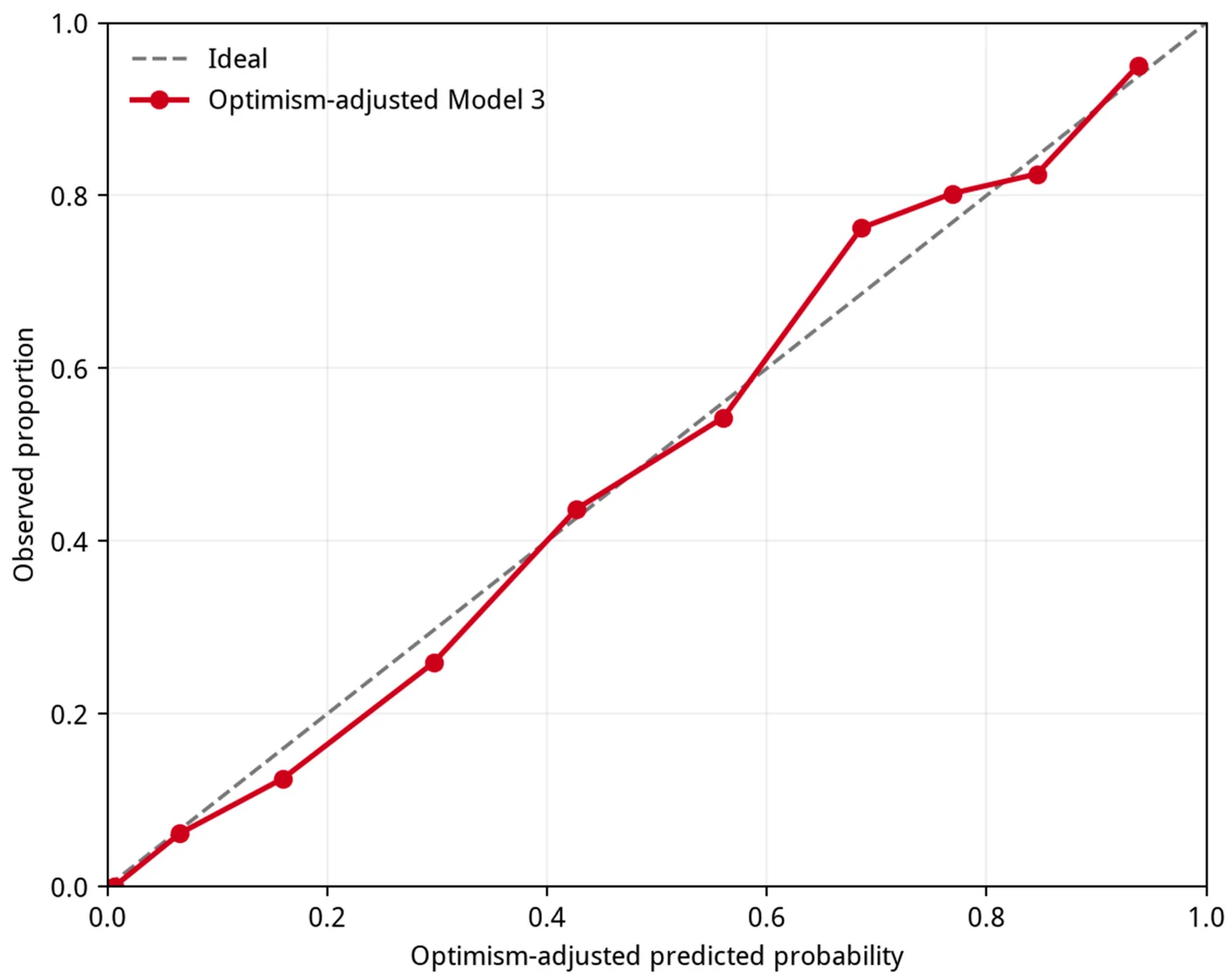
Calibration of the recalibrated version of Model 3. The original Model 3 linear predictor was transformed as logit(*p*_recalibrated) = 0.001 + 0.925 × logit(*p*_original), and probabilities were calculated from this transformed predictor. The corresponding adjusted intercept and coefficients are reported after Table 4. Predictions were grouped into deciles and compared with observed proportions. The diagonal represents perfect calibration. The figure therefore does not display predictions from the unadjusted equation alone.

**Figure 4:**
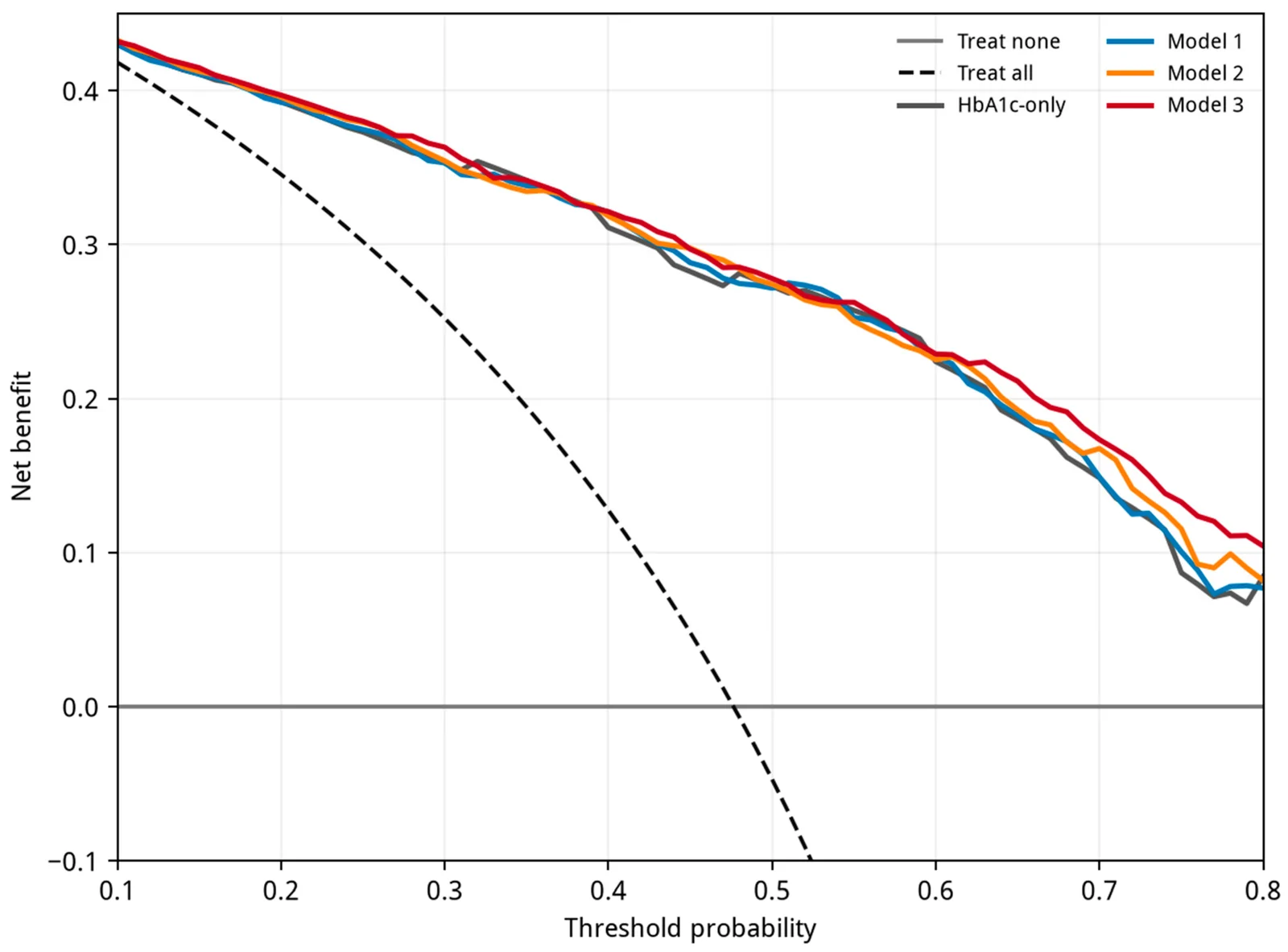
Exploratory decision-curve analysis evaluating the net benefit of intensified monitoring and/or treatment review for patients at risk of having HbA1c ≥7.0% at six-month follow-up. Curves compare the HbA1c-only model and Models 1–3 across threshold probabilities of 0.10–0.80, using the predicted probability of non-achievement (1 − p), where p denotes the predicted probability of HbA1c <7.0%. The black dashed curve represents intensified monitoring and/or treatment review for all patients (“Treat all”), whereas the horizontal gray line represents no such action for any patient (“Treat none”; net benefit = 0). False positives imply unnecessary additional monitoring or treatment review, and false negatives imply missed opportunities for intensified follow-up. The analysis is exploratory and requires external validation..

Table 5 compares apparent and optimism-corrected performance. Figure 3 shows calibration of the recalibrated version of Model 3, and Figure 4 presents exploratory decision-curve analysis.

## 4. Discussion

This study evaluated six-month HbA1c status in a single-center real-world cohort and directly addressed whether cardiorenal biomarkers and baseline treatment classes improve prediction beyond baseline HbA1c. The analysis yields a deliberately cautious conclusion: baseline HbA1c accounts for nearly all discrimination, while additional variables provide little internally validated incremental value.

The strong relationship between baseline and follow-up HbA1c is expected because both measurements reflect the same underlying glycemic process and the distance from the fixed 7.0% threshold. The baseline-by-follow-up table also shows that the original endpoint combined maintenance and new attainment. Reframing the primary endpoint as six-month glycemic status and restricting a sensitivity analysis to baseline HbA1c ≥ 7.0% avoids overstating the model as a predictor of treatment response [9,10,11,12,13,14].

Baseline insulin use should be interpreted as a marker of clinical context rather than a causal determinant of failure. Patients receiving insulin may have more advanced β-cell dysfunction, longer or more severe disease, prior treatment failure, or other unmeasured indications. Medication initiation, discontinuation, dose intensification, and adherence during the six-month interval were not systematically available. Fixing exposure at baseline does not create classic immortal-time classification, but unmeasured time-varying treatment causes exposure misclassification and substantial confounding by indication [15,16,17,18].

The TyG sign reversal was investigated rather than interpreted biologically. Conventional VIFs did not indicate severe multicollinearity, and the positive adjusted coefficient was stable in bootstrap resamples; however, TyG is mathematically related to glucose and triglycerides, and its adjusted sign depends on the covariance structure of the complete model. The negligible corrected performance gain and similar alternative fasting-glucose model argue against presenting TyG as a clinically useful independent predictor [19,20,21].

Cardiorenal and inflammatory biomarkers did not meaningfully improve validated discrimination or overall prediction error. This does not imply that these variables lack prognostic importance for cardiovascular or renal outcomes; it indicates only that they contributed little to this short-term HbA1c-status endpoint after baseline HbA1c was known [22,23,24,25,26,27,28].

The apparent therapeutic-block likelihood-ratio test was statistically significant, but likelihood improvement is not equivalent to clinically useful prediction. Model 3 had a higher AIC than the HbA1c-only model, its optimism-corrected AUC advantage was approximately zero, and decision curves showed, at most, marginal net-benefit differences. Accordingly, the model is not presented as a treatment algorithm or ready-to-use risk stratification system [8,29,30].

The practical implication is therefore not that clinicians should calculate the full equation but that a baseline HbA1c-centered assessment performs similarly in this dataset. Any future model intended to change care should incorporate dynamic treatment exposure, adherence, lifestyle and social determinants, define an actionable decision linked to predicted risk, and demonstrate improved patient outcomes in a prospective impact study [31,32,33,34,35,36,37,38].

Limitations include the retrospective, single-center design; the lack of external validation; the absence of a prospectively registered protocol; and the lack of direct comparison with an established model designed for an identical endpoint and population. Treatment changes, medication dose, adherence, dietary intake, physical activity, family history, detailed socioeconomic status, and ethnicity were unavailable, creating residual confounding and limiting transportability. The fixed 7.0% threshold does not capture individualized targets. The exact study period, source-population denominator, exclusion counts, permitted follow-up interval, and actual follow-up-time distribution are reported in Section 2.1 and Supplementary Figure S1 and must be verified against institutional records before submission. The apparent decision curve remains exploratory and must be externally validated.

Strengths include explicit separation of maintenance from new attainment, a common complete-case cohort for fair model comparison, an HbA1c-only benchmark, predictor blocks defined from clinical and conceptual considerations, bootstrap correction of optimism, reporting of calibration intercept and slope, collinearity and nonlinearity diagnostics, coefficient-stability assessment, the full prediction equation, and transparent acknowledgment of the model’s limited incremental clinical value.

## 5. Conclusions

Baseline HbA1c was the dominant predictor of six-month HbA1c status. After bootstrap correction, the full model did not improve discrimination over HbA1c alone.

The TyG coefficient reversal was not explained by severe VIF-defined multicollinearity, but its interpretation remains model-dependent. Baseline insulin use likely reflects confounding by indication and should not be treated as evidence of reduced therapeutic efficacy.

The current model has insufficient incremental value to support a treatment algorithm, online calculator, or routine clinical implementation. The complete equation is reported for reproducibility, not for clinical deployment.

External validation in ethnically and geographically diverse cohorts, collection of time-varying treatment and adherence data, and prospective assessment of decision and outcome impact are required before clinical use.

## Figures and Tables

**Table 1:** Baseline characteristics according to six-month HbA1c status.

Baseline Characteristic	HbA1c < 7.0% (*n* = 385)	HbA1c ≥ 7.0% (*n* = 423)	*p* Value
Demographic and clinical characteristics			
Age, years	64.04 ± 9.83	62.66 ± 10.20	0.050
Male sex, *n* (%)	213 (55.3)	217 (51.3)	0.283
Diabetes duration, years	6.00 (3.00–10.00)	7.00 (4.00–11.00)	0.149
Retired, *n* (%)	273 (70.9)	304 (71.9)	0.823
Baseline glycemic and metabolic profile			
HbA1c, %	7.00 ± 0.77	8.81 ± 1.62	<0.001
Fasting plasma glucose, mg/dL	164.24 ± 50.19	187.44 ± 61.38	<0.001
BMI, kg/m^2^	30.13 ± 5.10	30.68 ± 5.72	0.147
TyG index	9.32 ± 0.58	9.45 ± 0.67	0.003
LDL cholesterol, mg/dL	98.92 ± 32.18	97.45 ± 34.10	0.528
HDL cholesterol, mg/dL	45.43 ± 12.53	44.58 ± 12.36	0.331
Triglycerides, mg/dL	150.00 (106.00–198.00)	146.00 (100.50–208.00)	0.869
Renal, inflammatory, and hepatic profile			
eGFR, mL/min/1.73 m^2^	78.85 ± 19.48	79.32 ± 19.56	0.731
UACR, mg/mmol	2.20 (1.10–9.39)	2.20 (1.10–8.07)	0.534
FIB-4 index	1.28 ± 0.43	1.27 ± 0.38	0.853
CRP, mg/L	1.50 (1.00–3.70)	2.10 (1.00–3.95)	0.110
Baseline comorbidities and complications			
Hypertension, *n* (%)	221 (57.4)	228 (53.9)	0.353
Previous myocardial infarction, *n* (%)	13 (3.4)	15 (3.5)	1.000
Coronary heart disease, *n* (%)	49 (12.7)	56 (13.2)	0.911
Retinopathy, *n* (%)	81 (21.0)	90 (21.3)	1.000
Neuropathy, *n* (%)	112 (29.1)	113 (26.7)	0.500
Baseline antidiabetic treatment			
Insulin, *n* (%)	68 (17.7)	140 (33.1)	<0.001
Biguanides, *n* (%)	235 (61.0)	238 (56.3)	0.192
Sulfonylureas, *n* (%)	29 (7.5)	41 (9.7)	0.335
DPP-4 inhibitors, *n* (%)	60 (15.6)	77 (18.2)	0.370
GLP-1 receptor agonists, *n* (%)	71 (18.4)	61 (14.4)	0.147
SGLT2 inhibitors, *n* (%)	121 (31.4)	162 (38.3)	0.049

Data are mean ± standard deviation, median (interquartile range), or number (percentage). The table includes 385 participants with HbA1c < 7.0% and 423 with HbA1c ≥ 7.0%; variable-specific missingness is stated where applicable. *p* values are descriptive and were not used for predictor selection.

**Table 2:** Baseline-by-follow-up HbA1c status among 808 participants.

Baseline HbA1c Status	Follow-Up < 7.0%	Follow-Up ≥ 7.0%	Total
HbA1c < 7.0%	179	36	215
HbA1c ≥ 7.0%	206	387	593
Total	385	423	808

Values are numbers of participants. The row for baseline HbA1c < 7.0% represents maintenance or loss of pre-existing status; the row for baseline HbA1c ≥ 7.0% represents newly attained or persistently above-threshold status.

**Table 3:** Exploratory univariable logistic regression for six-month HbA1c < 7.0%.

Baseline Predictor	OR	95% CI	*p* Value
Age, per year	1.014	1.000–1.028	0.051
Female sex vs. male	0.851	0.645–1.122	0.252
Diabetes duration, per year	0.978	0.950–1.006	0.122
HbA1c, per 1% increase	0.189	0.148–0.241	<0.001
BMI, per 1 kg/m^2^ increase	0.981	0.957–1.007	0.150
TyG index, per one-unit increase	0.717	0.575–0.895	0.003
eGFR, per 1 mL/min/1.73 m^2^ increase	0.999	0.992–1.006	0.730
Log-transformed UACR	1.038	0.898–1.198	0.616
FIB-4 index, per one-unit increase	1.033	0.734–1.454	0.852
Log-transformed CRP	0.825	0.664–1.024	0.081
LDL cholesterol, per 1 mg/dL increase	1.001	0.997–1.006	0.529
HDL cholesterol, per 1 mg/dL increase	1.006	0.994–1.017	0.331
Triglycerides, per 1 mg/dL increase	0.999	0.998–1.001	0.341
Insulin use	0.434	0.311–0.604	<0.001
Biguanide use	1.218	0.920–1.613	0.169
Sulfonylurea use	0.759	0.462–1.248	0.277
DPP-4 inhibitor use	0.830	0.573–1.201	0.322
GLP-1 receptor agonist use	1.342	0.923–1.951	0.123
SGLT2 inhibitor use	0.738	0.552–0.988	0.041

OR, odds ratio; CI, confidence interval. UACR and CRP were transformed as ln(x + 1). Models included 808 participants, except UACR (*n* = 806). These analyses were descriptive and did not determine multivariable inclusion.

**Table 4:** Full multivariable logistic regression model for six-month HbA1c < 7.0%.

Baseline Predictor	Adjusted OR	95% CI	*p* Value
Age, per year	1.007	0.987–1.028	0.475
Female sex vs. male	0.857	0.588–1.249	0.422
Diabetes duration, per year	0.994	0.953–1.038	0.797
HbA1c, per 1% increase	0.184	0.143–0.238	<0.001
BMI, per 1 kg/m^2^ increase	0.995	0.959–1.032	0.783
TyG index, per one-unit increase	1.412	1.042–1.914	0.026
eGFR, per 1 mL/min/1.73 m^2^ increase	0.995	0.984–1.005	0.326
Log-transformed UACR	0.944	0.760–1.172	0.602
FIB-4 index, per one-unit increase	1.162	0.707–1.911	0.553
Log-transformed CRP	0.798	0.599–1.064	0.124
Insulin use	0.563	0.357–0.887	0.013
Biguanide use	1.291	0.876–1.902	0.197
Sulfonylurea use	0.550	0.288–1.052	0.071
DPP-4 inhibitor use	0.627	0.384–1.025	0.063
GLP-1 receptor agonist use	1.214	0.719–2.049	0.469
SGLT2 inhibitor use	0.804	0.537–1.202	0.287

The model used 806 complete cases. Binary indicators were coded 1 for female sex or medication use and 0 for male sex or non-use. Original prediction equation: logit(*p*) = 10.25260 + 0.00731 × age − 0.15447 × female − 0.00557 × diabetes duration − 1.69095 × HbA1c − 0.00509 × BMI + 0.34496 × TyG − 0.00544 × eGFR − 0.05754 × ln(UACR+1) + 0.15032 × FIB-4 − 0.22552 × ln(CRP+1) − 0.57407 × insulin + 0.25540 × biguanide − 0.59709 × sulfonylurea − 0.46672 × DPP-4 inhibitor + 0.19359 × GLP-1 receptor agonist − 0.21865 × SGLT2 inhibitor. The predicted probability is *p* = 1/[1 + exp(−logit(*p*))]. For the recalibrated predictions displayed in Figure 3, the original linear predictor was transformed as logit(*p*_recalibrated) = 0.001 + 0.925 × logit(*p*_original), giving the corresponding recalibrated equation: logit(*p*_recalibrated) = 9.484655 + 0.006762 × age − 0.142885 × female − 0.005152 × diabetes duration − 1.564129 × HbA1c − 0.004708 × BMI + 0.319088 × TyG − 0.005032 × eGFR − 0.053225 × ln(UACR+1) + 0.139046 × FIB-4 − 0.208606 × ln(CRP+1) − 0.531015 × insulin + 0.236245 × biguanide − 0.552308 × sulfonylurea − 0.431716 × DPP-4 inhibitor + 0.179071 × GLP-1 receptor agonist − 0.202251 × SGLT2 inhibitor. This recalibrated equation is provided solely to match Figure 3 and is not a new independently refitted model.

**Table 5:** Apparent and optimism-corrected performance in the common complete-case cohort (*n* = 806).

Model	AIC	Apparent AUC	Corrected AUC (95% CI)	Corrected Brier	Corrected Slope
HbA1c-only	722.03	0.869	0.868 (0.844–0.892)	0.146	0.996
Model 1	728.14	0.870	0.867 (0.842–0.889)	0.147	0.980
Model 2	730.39	0.875	0.867 (0.847–0.891)	0.148	0.950
Model 3	727.56	0.881	0.870 (0.848–0.893)	0.146	0.925

Decision-curve analysis showed only small and inconsistent net-benefit gains for Model 3 over HbA1c alone across thresholds from 0.10 to 0.80. In the baseline HbA1c ≥ 7.0% sensitivity cohort (*n* = 592 complete cases; 206 events), corrected AUC was 0.850 (95% CI 0.822–0.880) for HbA1c alone and 0.849 (95% CI 0.821–0.877) for Model 3; corrected calibration slopes were 1.000 and 0.883, respectively. This analysis confirms that additional predictors did not improve validated discrimination among participants who could newly attain the threshold.

## Data Availability

The original contributions presented in this study are included in this article. Further inquiries can be directed to the first authors.

## Supplementary Materials

The following supporting information can be downloaded at: https://www.mdpi.com/article/10.3390/diagnostics16193201/s1, Supplementary Table S1. Correlation and collinearity diagnostics. Supplementary Table S2. Sensitivity analysis restricted to baseline HbA1c ≥ 7.0% (*n* = 592 complete cases; 206 events). Supplementary Figure S1. Participant flow for outcome and complete-case analyses.

## Abbreviations

The following abbreviations are used in this manuscript:

AUC	Area under the curve
BMI	Body mass index
CI	Confidence interval
CRP	C-reactive protein
DPP-4	Dipeptidyl peptidase-4
eGFR	Estimated glomerular filtration rate
FIB-4	Fibrosis-4 index
GLP-1	Glucagon-like peptide-1
HbA1c	Glycated hemoglobin
HDL	High-density lipoprotein
IQR	Interquartile range
LDL	Low-density lipoprotein
OR	Odds ratio
ROC	Receiver operating characteristic
SD	Standard deviation
SGLT2	Sodium–glucose cotransporter-2
T0	Baseline assessment
T1	Six-month follow-up assessment
T2DM	Type 2 diabetes mellitus
TyG	Triglyceride–glucose index
UACR	Urinary albumin-to-creatinine ratio
