# Supplementary material for: Prediction of Six-Month Glycemic Status in Type 2 Diabetes: Development and Internal Validation of a Multivariable Model
Source: Diagnostics (Basel). 2026 Oct 2;16(19):3201. doi: 10.3390/diagnostics16193201 (PMC13664384; doi:10.3390/diagnostics16193201)
Supplement: Supplementary file 1 [file diagnostics-16-03201-s001.zip › diagnostics-4535065-supplementary.pdf]

Supplementary Table S1. Correlation and collinearity diagnostics.

Diagnostic	Value
Pearson r: HbA1c–fasting glucose	0.448
Pearson r: HbA1c–triglycerides	0.119
Pearson r: HbA1c–TyG	0.302
Pearson r: fasting glucose–TyG	0.548
Pearson r: triglycerides–TyG	0.795
VIF: HbA1c	1.18
VIF: TyG	1.12
Maximum predictor VIF	1.33
TyG coefficient positive in bootstrap samples	99.0%

Supplementary Table S2. Sensitivity analysis restricted to baseline HbA1c ≥7.0% (n=592 complete cases; 206 events).

Model	Corrected AUC (95% CI)	Corrected Brier	Corrected intercept	Corrected slope
HbA1c-only	0.850 (0.822–0.880)	0.150	−0.002	1.000
Model 3	0.849 (0.821–0.877)	0.151	0.004	0.883

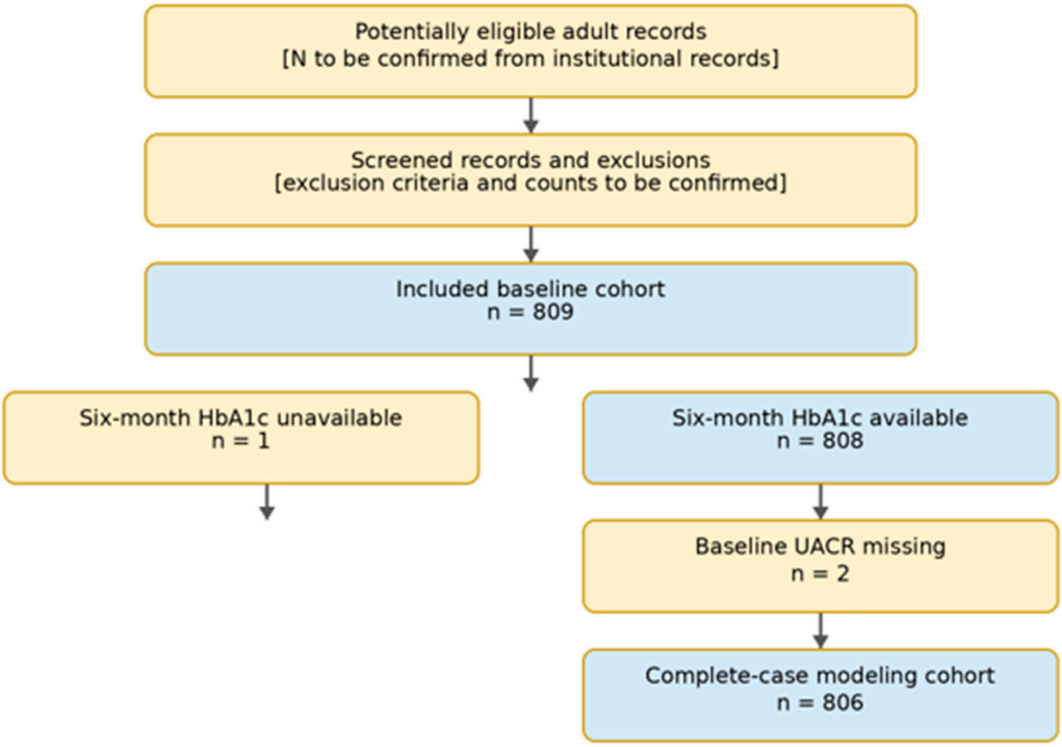

Supplementary Figure S1. Participant flow for outcome and complete-case analyses.
